# A one-year follow-up case series on gait analysis and patient-reported outcomes for persons with unilateral and bilateral transfemoral amputations undergoing direct skeletal fixation

**DOI:** 10.1186/s12984-024-01509-4

**Published:** 2024-11-29

**Authors:** Diana Toderita, Tiereny McGuire, Alice M. Benton, Charles Handford, Arul Ramasamy, Paul Hindle, Anthony M. J. Bull, Louise McMenemy

**Affiliations:** 1 Department of Bioengineering, Imperial College London, London, UK; 2 Academic Department of Military Trauma and Orthopaedics, Birmingham, UK; 3 St. Paul’s Hospital, Vancouver, Canada; 4 University of British Columbia, Vancouver, Canada

**Keywords:** Osseointegrated prostheses, Transfemoral amputation, Gait analysis, Musculoskeletal modelling

## Abstract

**Background:**

Direct skeletal fixation, a surgical technique enabling the attachment of an external prosthesis directly to the bone through a percutaneous implant, offers an enticing solution for patients with lower limb amputations facing socket-related issues. However, understanding of its impact on musculoskeletal function remains limited.

**Methods:**

This study compares pre- and 1-year post-osseointegration surgery outcomes, focusing on patient-reported measures and musculoskeletal system function during level-ground walking. Two participants with unilateral transfemoral amputations and two participants with bilateral transfemoral amputations completed the questionnaire for transfemoral amputations (Q-TFA) and underwent gait analysis. Musculoskeletal modelling simulations were conducted.

**Results:**

Results showed improved Q-TFA scores for all participants. Participants showed reduced amputated limb peak hip extension angles, flexion torques and contact forces at the push-off phase of the gait cycle. Post-operatively, hip adduction angles and abduction moments increased, indicating more natural gait patterns. Whilst one participant demonstrated increased post-operative walking speed, others walked more slowly.

**Conclusions:**

The study revealed diverse adaptation patterns after one year in individuals with transfemoral amputations transitioning to bone-anchored prostheses. Additional longer-term data is necessary to enable generalization and clinical implications of these results.

## Background

During the 2003–2014 Afghanistan conflict, there were 265 UK Armed Forces Service Personnel who suffered major joint amputations with a recorded total of 416 amputations [1]. The most common form of amputation was at the transfemoral level, affecting 153 individuals [1]. Although these severely disabling injuries have been successfully treated with advanced rehabilitation and modern prostheses, traditional prosthetic sockets were not the solution for all persons with limb loss. Some individuals report complications related to their stump, including skin breakdown, discomfort, and balance issues, which can lead to poor physical capabilities and eventual prosthetic abandonment [2–4]. These challenges are exacerbated for individuals with short stumps, commonly seen in blast injury patients [5], and those with surgical excision of heterotopic ossification [6], further complicating the fitting of traditional sockets. For such individuals, osseointegration presents a promising alternative fixation method. Osseointegration involves directly fixing the prosthesis to the bone through a percutaneous implant [7], eliminating the need for a prosthetic socket. Previous research on the initial UK military cohort undergoing osseointegration presented improved physical activity levels at 2-year post-surgery compared to pre-operative measures, with minimal complications [8].

After limb loss, individuals adapt their walking pattern to accommodate for the absence of muscle, joints, and physiological insertion points. The choice of prosthetic type can influence these adjustments [9–12]. Whilst these compensatory strategies are necessary for effective ambulation, they may lead to musculoskeletal issues. Studies indicate that individuals with unilateral transfemoral amputations (UTF) are at higher risk of knee osteoarthritis in the non-amputated limb [4, 13, 14], as well as hip osteoarthritis in both the intact and amputated limbs than persons without limb loss [14]. This increased risk may be attributed to abnormal joint loading patterns [15]. Whilst there is some understanding of these issues among individuals using socket-suspended prostheses, limited knowledge exists regarding those with bone-anchored (osseointegration) prostheses. Previous research has shown slightly greater hip cartilage abrasion in socket users compared to bone-anchored prosthesis users, with the amputated side hip exhibiting higher abrasion than the intact limb in socket users [16]. However, additional research is needed to understand this phenomenon further.

The relationship between mechanical loading and joint degeneration progression in individuals with bilateral transfemoral amputations (BTF) remains unclear. Previous studies have examined the functional abilities for BTF with prosthetic sockets, showing slower walking speeds and cadence, as well as higher oxygen cost compared to individuals without limb loss [17]. Following amputation, the loading of the musculoskeletal system is altered, resulting in greater hip joint moments and power than those of persons without limb loss [11, 18]. Prosthetic options for BTF include full-length articulated prostheses, which feature microprocessor-controlled knees and dynamic response feet, as well as non-articulated stubbies. Research has indicated that BTF using full-length articulated prostheses experience increased hip joint contact forces and hip flexor muscle forces compared to persons without limb loss, while similar hip joint contact forces have been observed between BTF using stubbies and persons without limb loss [11]. Although these findings have been documented for BTF with prosthetic sockets, to our knowledge, there has been no investigation into the pre- and post-operative status of BTF who have experienced socket-related issues and required osseointegration surgery.

Musculoskeletal modelling is a non-invasive robust biomechanical tool used to explore joint contact forces for individuals with transfemoral amputations [11, 19, 20], those with transtibial amputations [21], and individuals without limb loss [22, 23]. Understanding joint contact forces is important as they relate to joint health, and can be indicative of osteoarthritis development and progression [24, 25]. Several gait analysis studies have investigated how UTF musculoskeletal system loading changes after osseointegration during gait [26, 27] and stand-to-sit [28], but none have focused on BTF. As osseointegration is a relatively recent surgical intervention, more data are needed to develop a comprehensive understanding of its outcomes.

This prospective study aims to compare the pre-operative and 1-year post-operative patient-reported outcomes and musculoskeletal system function during walking for UTF and BTF from the second cohort of UK military amputees who experienced socket related issues with traditional prosthetics. This study provides a foundation for understanding the clinical outcomes of osseointegration in military traumatic amputees who are unable to use prosthetic sockets. These findings can support clinicians in making evidence-based decisions regarding osseointegration for transfemoral amputees, ultimately promoting optimal patient outcomes.

## Methods

Ethical approval was obtained from the Imperial College Science, Engineering and Technology Research Ethics Committee (Reference 22IC7601, 20IC6268). Written informed consent was obtained from all study participants. Participants sustained transfemoral traumatic amputations, with one (BTF2) also having an upper limb amputation above the left elbow (Table 1). Pre-operatively, all participants used ischial containment prosthetic sockets. They underwent implantation with the Australian Osseointegration Group of Australia-Osseointegration Prosthetic Limb (OGAP-OPL) prosthesis and received a standardised rehabilitation program at the Defence Medical Rehabilitation Centre United Kingdom post both initial amputation and osseointegration surgery. Post-operatively, participant UTF2 developed a neuroma in the residual limb, whereas the other participants did not report any complications.

**Table 1:** Participant demographics

Participant	Age (years)	Sex	Pre-injury height (m)	Pre-op adjusted mass(kg)	1-year post-op adjusted mass (kg)	Pre-op residual limb length (cm)	Time since amputation (years)	Prostheses	Reason for osseointegration
UTF1	33	Male	1.80	91.3	92.3	40	7	C-leg, Triton	Stump pain related to socket use
UTF2	44	Male	1.71	87.4	77.4	40	8	Genium X3, Triton Low Profile	Stump pain related to socket use; poor socket fit
BTF1	43	Male	1.80	96.2	91.0	39 (left), 25 (right)	11	Stubbies	Stump pain related to socket use; wound development on stump
BTF2	46	Male	1.74	102.9	100.9	41 (left), 38 (right)	12	Genium X3, Triton Low Profile, walking stick	Back and stump pain related to socket use

Age, time since amputation at 1-year follow-up. Mass adjusted for amputation according to literature [29]

Gait data were collected using a 10-camera VICON optoelectronic based motion capture system (VICON 2.11, Oxford Metrics Group, UK) at an acquisition rate of 120 Hz and two force plates (Kistler Type 9286B, Kistler Instrumente AG, Winterthur, Switzerland) at an acquisition rate of 1200 Hz. Retro-reflective markers were attached to anatomical landmarks of the lower limbs following previous studies [11, 30, 31]. The marker set used was the same for participants with full-length articulated prostheses and for the participant with stubby prostheses, following previous literature [11]. For the stubby prostheses, knee markers were placed on the medial and lateral sides of the pylon, below the prosthetic socket and below the connector post-operatively. One static and three self-selected walking speed gait trials with complete foot placement on the force plates were collected for all participants. The walking stick used by BTF2 was placed outside of the force plates in all trials. The motion data were processed in VICON Nexus (VICON 2.11, Oxford Metrics Group, UK) and MATLAB (The Mathworks Inc., Natick, USA). The gait cycle events (heel strike, toe off, consecutive ipsilateral heel strike) were identified using a 25 N force threshold. The marker trajectories and force plate data were filtered using a zero phase-lag, fourth order Butterworth filter with 6 Hz cut-off frequency [21, 32, 33].

Freebody [22] was used to perform inverse kinematics, inverse dynamics and musculoskeletal modelling simulations following previous methodology for the intact [23] and amputated limb [11, 20]. In the pre-operative simulations, the stump, prosthetic liner, socket, and connector were combined and modelled as a single rigid segment representing the thigh. In the post-operative model, the prosthetic socket was excluded, and the thigh segment was modelled as a rigid segment consisting of the stump and the implant. For both pre- and post-operative cases, the interface between the stump and prosthesis was not modelled in detail, and the femoral segment was assumed to be rigid throughout. For stubby prostheses, the shank segment is represented by the pylon connecting the foot to the thigh segment, where the connection between the pylon and thigh segment acts as locked joint. In the musculoskeletal model, torque values were measured at the midpoint between the medial and lateral epicondyles equivalents for the prosthetic knee, and between the medial and lateral malleoli equivalents for the prosthetic ankle. Since no movement occurs for stubbies at the locked joint, the torque created at the ankle joint is transmitted directly to the locked joint and subsequently to the hip. The amputee musculoskeletal model with full-length articulated and stubby prostheses has been validated using electromyography measurements [20]. The selection of the anatomical dataset for both the intact and amputated limb models was based on the closest anthropometry similarity [20, 30]. The same anatomical dataset was used for each model pre- and post-operatively.

The following biomechanical parameters were analysed: the percentage of the gait cycle spent in the stance phase, intact limb step length (calculated as the anterior-posterior distance between the prosthetic heel marker location at the prosthetic limb heel strike and the intact heel marker at the intact limb heel strike), prosthetic limb step length (calculated as the anterior-posterior distance between the intact heel marker at the intact limb heel strike and the prosthetic heel marker location at the prosthetic limb heel strike), stride length, step width (calculated as the medial-lateral distance between the intact heel marker at the intact limb heel strike and prosthetic heel marker at the prosthetic limb heel strike), stance width in the static trial (calculated as the medial-lateral distance between the intact heel marker and prosthetic heel marker in the static trial), cadence, walking speed, net ground reaction forces, joint angles, joint torques, joint contact forces, joint impulse (calculated as the area under the contact force curve), and normalised joint impulse (calculated as the area under the contact force curve divided by the walked distance).

The patient focused outcomes were assessed using the questionnaire for persons with transfemoral amputations (Q-TFA) [34]. Comparisons were made between pre- and 1-year post-operative measures, where participants acted as their own controls using a repeated measures design. The results are presented in a case study manner.

## Results

### UTF

Table 2 presents the UTF Q-TFA questionnaire results and temporospatial characteristics. Both participants showed improvements across all Q-TFA scales. The problem score for UTF1 was invalid due to an insufficient number of items answered. The post-operative Q-TFA global score showed a 150% increase for UTF1 and a 50% increase for UTF2 compared to the pre-operative values. In comparison to pre-operative values, UTF1 showed a post-operative 24% increase in intact limb stride length, a 24% increase in prosthetic limb stride length, a 4% increase in cadence, and a 23% increase in walking speed. The step width decreased by 21% compared to pre-operative levels. Conversely, UTF2 demonstrated a post-operative decrease in intact limb stride length by 6%, prosthetic limb stride length by 7%, cadence by 9%, and walking speed by 15% relative to pre-operative values. UTF2 showed a 5% increase in intact limb post-operative percentage stance time compared to pre-operative measurements.

**Table 2:** UTF Q-TFA results and gait analysis temporospatial characteristics

Q-TFA results								
Scale score	UTF1				UTF2			
	Pre-op		1-year post-op		Pre-op		1-year post-op	
Prosthetic use	0		100		51.61		70.97	
Prosthetic mobility	30		93.89		55.56		83.33	
Global	33.33		83.33		33.33		50	
Problem	Invalid		3.33		53.33		20.83	
*Gait analysis temporospatial characteristics*								
Parameter	UTF1				UTF2			
	Pre-op		1-year post-op		Pre-op		1 -year post-op	
	Intact limb	Prosthetic limb	Intact limb	Prosthetic limb	Intact limb	Prosthetic limb	Intact limb	Prosthetic limb
Stance time (% gait cycle)	65.51	63.05	65.27	58.59	65.49	61.93	68.61	60.89
Step length (m)	0.57	0.62	0.79	0.72	0.78	0.64	0.66	0.67
Stride length (m)	1.24	1.22	1.54	1.51	1.46	1.46	1.37	1.36
Step width (m)	0.19		0.15		0.24		0.25	
Stance width in the static trial (m)	0.24		0.20		0.35		0.27	
Cadence (steps/min)	103.23		107.30		102.90		93.54	
Walking speed (m/s)	1.10		1.35		1.25		1.06	

A score of 100 indicates the best possible score for the Prosthetic use, Prosthetic mobility, and Global scales. A score of 0 indicates the best possible score for the Problem scale

**Fig. 1:**
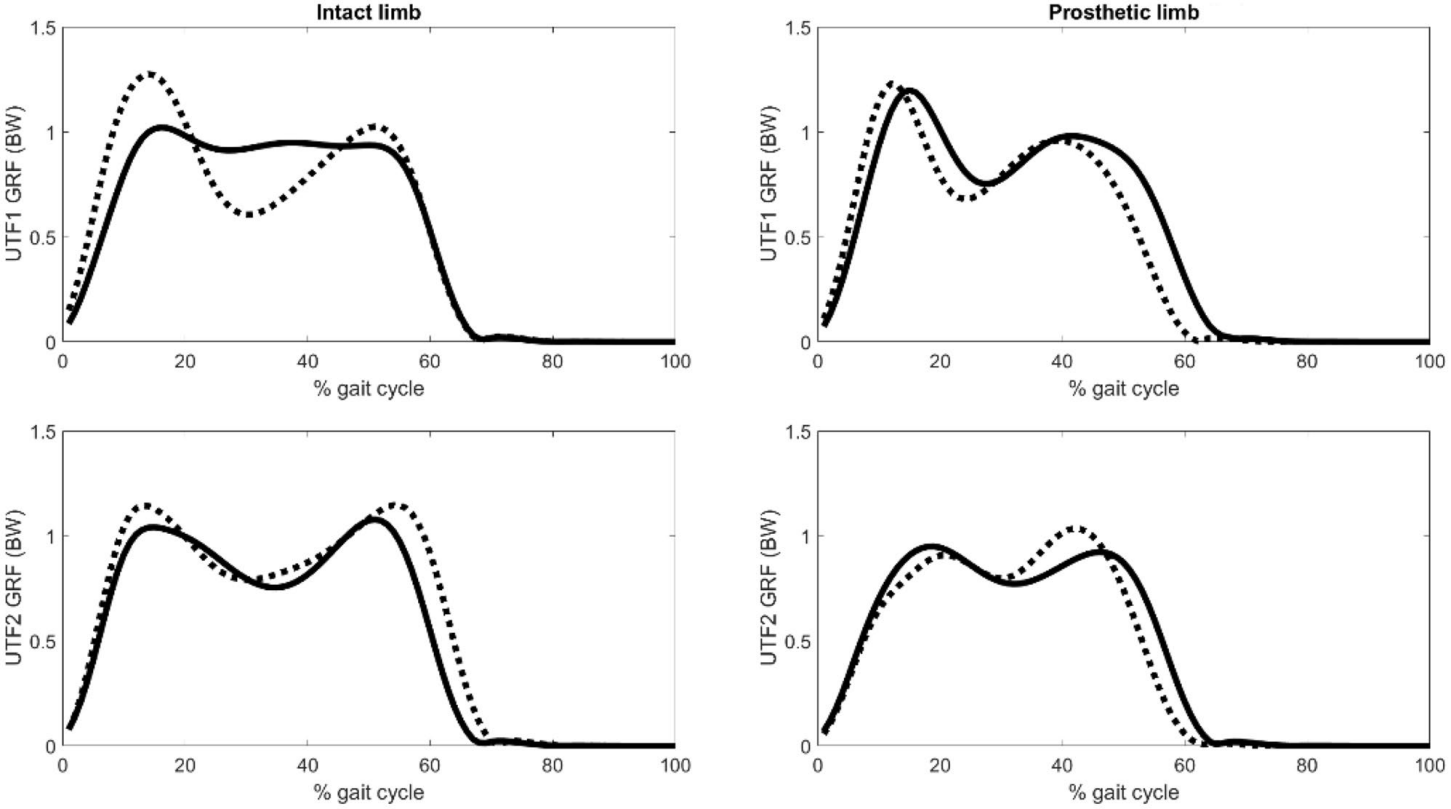
Pre-operative (solid), 1-year post-operative (dashed) ground reaction forces (GRF) for UTF1 (top) and UTF2 (bottom)

Figure 1 presents the UTF ground reaction forces over the course of the gait cycle from 0% at heel strike to 100% at the consecutive ipsilateral heel strike. Relative to pre-operative values, UTF1 showed a 23% increase in intact limb first peak ground reaction force and UTF2 demonstrated a 9% increase in intact limb first peak ground reaction force.

**Fig. 2:**
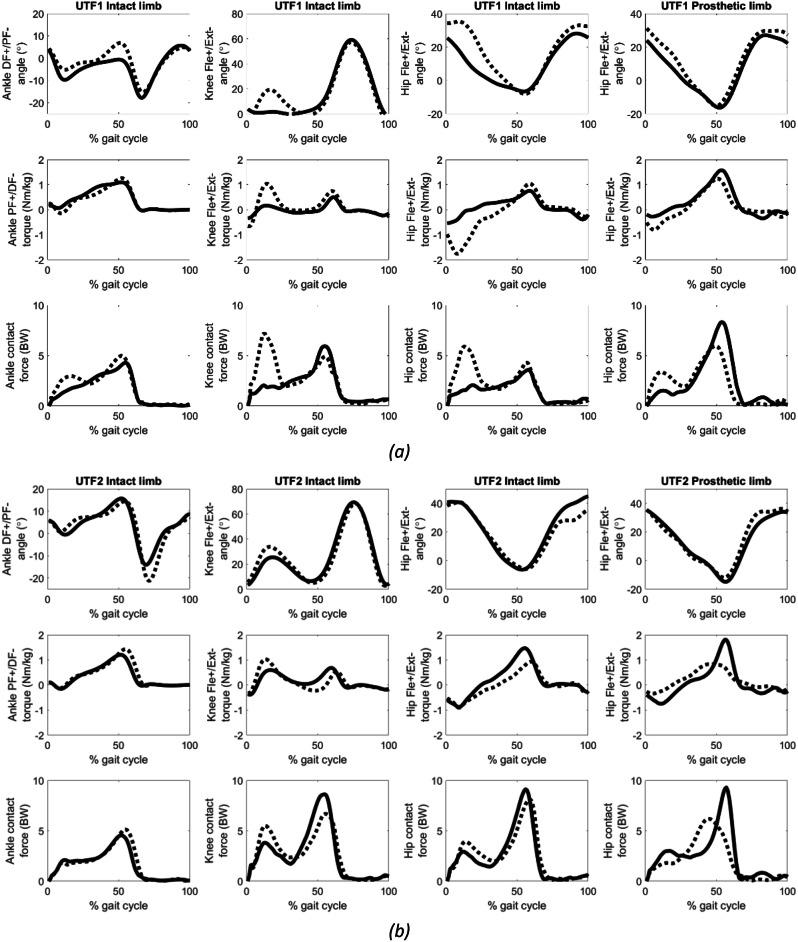
Pre-operative (solid), 1-year post-operative (dashed) joint angles, torques, contact forces for **(a)** UTF1, **(b)** UTF2

Figure 2 illustrates the joint angles, torques, and contact forces. There were notable post-operative changes: the peak ankle plantarflexion decreased by 14% for UTF1 and increased by 50% for UTF2. Both participants exhibited increased intact knee flexion in early stance, with UTF1 showing a 299% increase and UTF2 a 32% increase. The first peak intact knee contact force increased by 213% for UTF1 and by 48% for UTF2, the second peak intact knee contact force decreased by 17% for UTF1 and by 13% for UTF2, and the normalised impulse increased by 2.5% for UTF1 and by 11% for UTF2 (Table 3).

Following osseointegration, both participants presented decreased peak hip extension angles (by 8% for UTF1 and by 20% for UTF2) and flexion torques (by 24% for UTF1 and by 54% for UTF2) for the amputated limb at the push-off phase of the gait cycle. The second peak of the amputated limb hip joint contact forces decreased by 31% for UTF1 and by 33% for UTF2. Additionally, the amputated side hip impulse decreased by 24% for UTF1 and by 7% for UTF2. The intact side hip impulse increased by 19% for UTF1 and by 8% for UTF2 (Table 3).

**Table 3:** UTF joint contact force impulse

Parameter	UTF1				UTF2			
	Pre-op		1-year post-op		Pre-op		1-year post-op	
	Intact limb	Prosthetic limb	Intact limb	Prosthetic limb	Intact limb	Prosthetic limb	Intact limb	Prosthetic limb
Intact ankle impulse (BW.s)	155.51	N/A	185.44	N/A	157.23	N/A	168.22	N/A
Normalised intact ankle impulse (BW.s/m)	125.61	N/A	120.51	N/A	106.54	N/A	123.26	N/A
Intact knee impulse (BW.s)	198.19	N/A	249.84	N/A	272.77	N/A	284.00	N/A
Normalised intact knee impulse (BW.s/m)	158.35	N/A	162.33	N/A	185.08	N/A	206.19	N/A
Hip impulse (BW.s)	143.54	225.78	210.85	212.81	244.76	244.89	250.68	211.31
Normalised hip impulse (BW.s/m)	114.81	186.73	136.96	141.34	166.07	167.81	180.71	155.56

### BTF

Table 4 presents the BTF Q-TFA questionnaire results and temporospatial characteristics. Both participants showed improvements across all Q-TFA scales. The prosthetic mobility score for BTF2 was invalid due to an insufficient number of items answered. The post-operative Q-TFA global score showed an 83% increase for BTF1 and a 100% increase for BTF2 compared to pre-operative values. BTF1 showed a post-operative decrease in stride length by 29%, cadence by 8%, walking speed by 32%, and an increase in step width by 16%. BTF2 showed a post-operative decrease in stride length by 9%, walking speed by 6%, and a 10% decrease in step width.

**Table 4:** BTF Q-TFA results and gait analysis temporospatial characteristics

Q-TFA results								
Scale score	BTF1				BTF2			
	Pre-op		1-year post-op		Pre-op		1-year post-op	
Prosthetic use	32.26		51.61		23.04		90.32	
Prosthetic mobility	77.22		85.56		43.33		Invalid	
Global	50		91.67		33.33		66.66	
Problem	33.33		0.83		84.48		27.59	
*Gait analysis temporospatial characteristics*								
Parameter	BTF1				BTF2			
	Pre-op		1-year post-op		Pre-op		1-year post-op	
	Left limb	Right limb	Left limb	Right limb	Left limb	Right limb	Left limb	Right limb
Stance time (% gait cycle)	62.83	60.85	58.60	63.25	71.03	67.10	67.53	64.72
Stride length (m)	0.96	0.94	0.74	0.67	0.96	0.95	0.90	0.86
Step length (m)	0.43	0.53	0.35	0.39	0.46	0.50	0.43	0.47
Step width (m)	0.25		0.29		0.30		0.27	
Stance width in the static trial (m)	0.37		0.31		0.36		0.28	
Cadence (steps/min)	107.73		98.63		79.45		81.53	
Walking speed (m/s)	0.84		0.57		0.64		0.60	

A score of 100 indicates the best possible score for the Prosthetic use, Prosthetic mobility, and Global scales. A score of 0 indicates the best possible score for the Problem scale

**Fig. 3:**
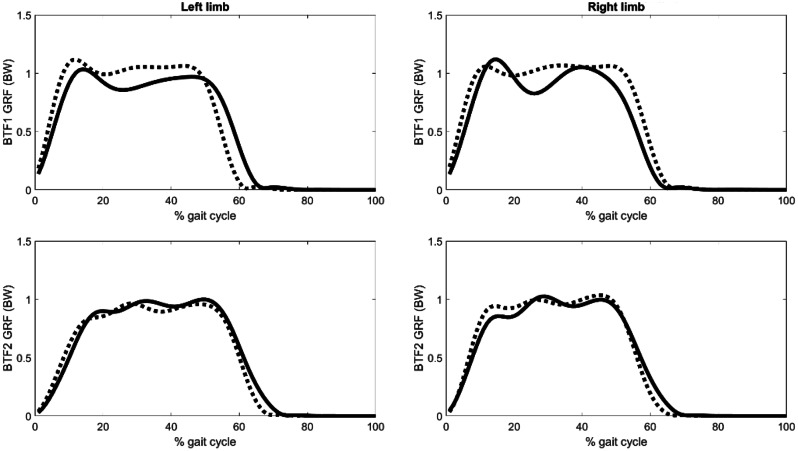
Pre-operative (solid), 1-year post-operative (dashed) ground reaction forces (GRF) for BTF1 (top) and BTF2 (bottom)

**Fig. 4:**
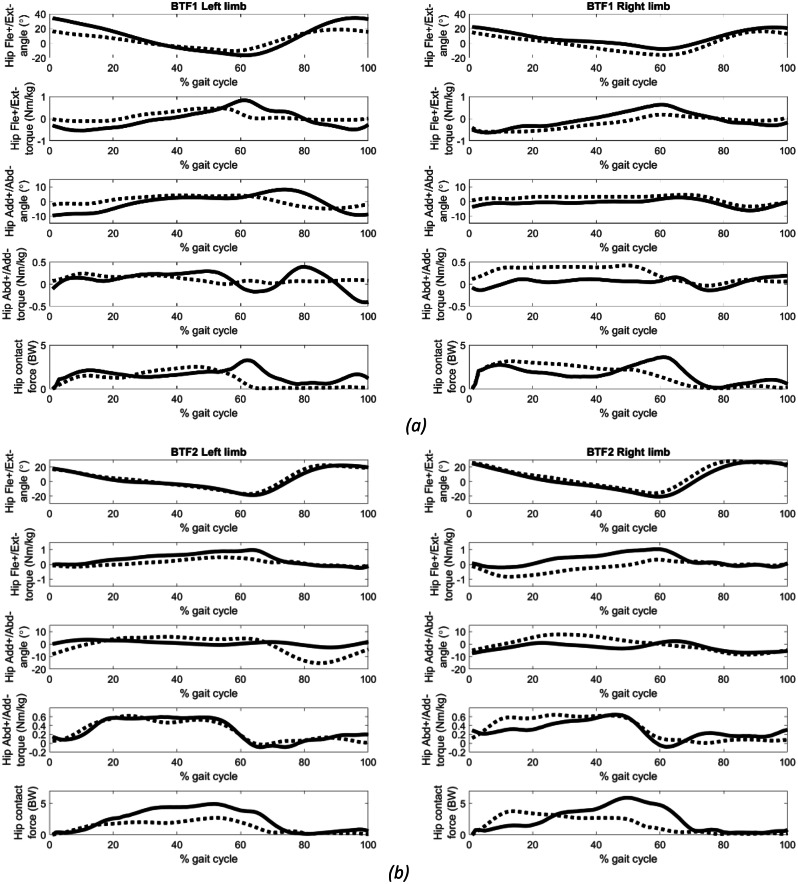
Pre-operative (solid), 1-year post-operative (dashed) hip angles, torques, contact forces for **(a)** BTF1, **(b)** BTF2

Figure 3 presents the GRF and Figure 4 illustrates the angles, torques, and contact forces at the hip joints. With the exception of the right limb of BTF2, there was a post-operative increase in the peak hip extension angle for all cases. The right limb of BTF2 showed notable improvements in hip adduction angles during stance. At push-off, there was a decrease in peak hip flexion torques and contact force for all limbs post-operatively. The normalised hip impulse decreased post-operatively for all limbs, except for the right limb of BTF2, which showed an increase (Table 5).

**Table 5:** BTF hip loading dose

Parameter	BTF1				BTF2			
	Pre-op		1-year post-op		Pre-op		1-year post-op	
	Left limb	Right limb	Left limb	Right limb	Left limb	Right limb	Left limb	Right limb
Hip impulse (BW.s)	157.48	169.99	107.35	160.14	234.77	235.23	126.16	163.51
Normalised hip impulse (BW.s/m)	164.25	180.35	145.88	240.19	243.48	246.23	139.30	191.27

## Discussion

This prospective study investigated UTF and BTF musculoskeletal system function and loading with traditional socket-anchored prostheses and at 1-year post-osseointegration surgery with bone-anchored prostheses.

### UTF

Pre-operatively, UTF1 had a low Q-TFA Prosthetic Use score, mostly relying on a wheelchair for mobility, whereas UTF2’s score indicated more frequent prosthesis use. Individuals accustomed to socket-suspended prostheses develop specific walking patterns that require time to re-adjust when transitioning to bone-anchored prostheses. This process can potentially span more than one year, as individuals aim to regain confidence in their walking. Interestingly, UTF1 transitioned more smoothly to walking with bone-anchored prostheses compared to UTF2, who was already accustomed to walking on sockets. This was evidenced by UTF1’s faster post-operative walking speed and higher post-operative Q-TFA score compared to UTF2. This difference underscores the individualized nature of adaptation, with prior mobility status potentially influencing adjustment ease.

UTF1’s post-operative increase in walking speed was achieved through an increase in stride length, rather than cadence, aligning with existing literature [19, 35]. This increase in walking speed led to higher ground reaction forces [36] compared to pre-operative measures. Nonetheless, post-operatively, there was an improvement in symmetry in ground reaction forces between limbs. Additionally, the intact limb exhibited a more natural loading profile, displaying a two-peak pattern and natural roll-over function of the intact foot. This has likely been facilitated by the intact knee flexion in early stance, a movement not observed pre-operatively, where the intact knee remained fully extended for potential stability while the prosthetic limb was in swing. UTF1 maintained consistent stance phase duration in the intact limb gait cycle, while the prosthetic limb stance duration decreased post-operatively, aligning with existing literature [37]. This shift may be attributed to the faster walking speed [36].

Individuals with transfemoral amputations often widen their step width during gait to better mechanically recruit the abductor muscles, enhance stability and power production capabilities [17, 38], also accommodating the bulky socket. It was expected that after socket removal, the step width would decrease [16], as seen in UTF1. However, the step width remained similar for UTF2, possibly indicating confidence issues with the prosthesis. The stance width during the static trial decreased for all participants after osseointegration, as the removal of the bulky prosthetic socket eliminated the need for a larger stance width while standing. The balance between the absence of the prosthetic socket and compensatory movements affecting frontal alignment likely varies between participants. Previous research found no significant differences in step width during walking between baseline and bone-anchored prostheses at 1-year follow-up [37].

UTF often excessively plantar flex the intact ankle [18], suggesting a vaulting mechanism [39] adopted to create clearance for the prosthetic toe in mid-swing to prevent tripping [40]. UTF1 showed a significant reduction in intact ankle plantar flexion post-osseointegration, also seen in the literature [41], leading to a more natural gait pattern and decreased functional demand on the ankle, as evidenced by the reduced normalised intact ankle impulse. Conversely, UTF2 exhibited an increase in intact ankle plantar flexion post-operatively, suggesting a greater functional demand of the intact limb, which aligns with the increased intact limb stance time and decreased prosthetic limb stance time.

The post-operative adaptation observed in UTF2 is further supported by an increased first peak of the ground reaction force and decreased first peak of the prosthetic limb ground reaction force compared to pre-operative measurements, potentially linked to a neuroma developed by UTF2. Pain management may have driven the offloading of the prosthetic limb to alleviate discomfort. Despite the slower walking pace and different loading pattern, UTF2 perceived the osseointegration surgery beneficial for daily functionality, highlighting the limitations of socket-suspended prostheses.

Post-operatively, both participants exhibited a decrease in prosthetic limb hip extension compared to pre-operative measures. Traditionally, persons with transfemoral amputations rely on increased hip extension with socket-suspended prostheses to propel the prosthetic limb forward, placing a greater burden on the hip flexor muscles [42, 43]. However, with bone-anchored prostheses, individuals gain greater control over their prosthetic limb and eliminate the need for the heavy prosthetic socket, thereby eliminating the need for increased hip extension. The reduction in prosthetic limb hip extension subsequently leads to decreased hip flexion moments during push-off and second peak of the hip joint contact force [27]. The lighter thigh segment, resulting from the removal of the prosthetic socket, also likely contributed to reduced hip joint contact forces. Furthermore, the prosthetic side hip joint impulse decreased post-operatively, while an increase was observed on the intact side for both participants. This increase might be attributed to UTF1’s increased walking speed and UTF2’s compensatory movement strategy, which relies predominantly on the intact limb.

Both participants exhibited similar post-operative changes in the loading of the intact knee, characterized by an increase in the first peak of the intact knee contact force and a decrease in the second peak, consistent with previous findings [27]. The increase in the first peak might be attributed to the higher intact limb ground reaction force and improved ability to flex the intact knee in early stance. The intact knee impulse per gait cycle increased post-operatively for both participants. However, after normalisation to walked distance, the normalised impulse was similar between time points for UTF1, but remained higher post-operatively for UTF2, indicating increased reliance on the intact limb, as reported anecdotally by the participant.

The study suggests that osseointegration may improve hip joint loading on the amputated side but could potentially increase loading on the intact knee and hip. Abnormally increased joint loading is associated with tissue degeneration and onset of osteoarthritis [21, 24, 25, 44, 45], but the long-term implications of these changes at 1-year remain unclear. Osteoarthritis is a complex condition influenced by both mechanical and biological factors [46, 47], making it challenging to draw definitive conclusions from these findings alone. Additional follow-up data and a greater sample size are necessary to enable generalization and clinical implications of these results. Given the variability among participants in anatomy, injury, and mobility status, cases studies are crucial for a comprehensive understanding of the outcomes of this novel surgical approach.

### BTF

Post-operatively, decreased walking speeds were observed in both participants with bilateral transfemoral amputations, consistent with a previous study [37]. This could be attributed to the need for an extended adaptation and training duration beyond one year, particularly considering that participants had used prosthetic sockets for an extended period. It is possible that complete confidence in the prostheses had not yet been achieved, as evidenced by the fact that BTF2 utilized an upper limb prosthesis and walking aid post-operatively, but not pre-operatively, finding them beneficial for balance. Despite lower walking speeds and cadence with osseointegration, participants perceived increased functional abilities post-operatively based on the Q-TFA results. Prior research has similarly noted improvements in Q-TFA scores among individuals with transfemoral amputations one year after undergoing osseointegration surgery [48–50]. Whilst gait analysis provides valuable insights into musculoskeletal loading, it alone does not fully capture real-world functionality beyond the laboratory. Moreover, the self-selected walking speed may not reflect the participants’ fastest pace, as they may have opted for the most energy-efficient speed.

Except for BTF1’s right limb, BTF1’s left limb and BTF2 exhibited trends similar to UTF participants, with reduced hip extension angles, flexion torques, and contact forces post-operatively, consistent with previous literature [27]. However, BTF2 used a stick on their right side, potentially impacting their walking pattern and hip joint forces [51]. BTF1’s short right limb showed increased hip extension angles post-operatively despite minimal extension pre-operatively, in contrast to the left limb and BTF2. The prosthetic socket restricted sagittal plane hip range of motion [52]. Both participants showed post-operative improvements in sagittal plane hip range of motion symmetry, particularly notable for BTF1. Muscle imbalance due to BTF1’s short residuum and weak adductor muscles led to a misaligned abducted position. Following surgery, BTF1 achieved improved limb alignment and displayed increased adduction angles and abduction moment during stance post-operatively, resembling more natural gait patterns [16, 53]. The improved reliance on the right limb during gait is also evidenced by the increased ground reaction forces and prolonged time spent in its stance phase post-operatively, allowing for improved weight distribution onto the right limb, indicated by the increased right hip normalised impulse post-operatively.

## Limitations

There are limitations to this study. The small sample size restricts the generalizability of the descriptive findings. However, given that osseointegration is a novel surgical procedure and considering the significant anatomical variability among persons with lower limb amputations [20], it is essential to derive comprehensive insights from case series to better understand the clinical implications of this surgery. Larger cohort studies are required to ensure that the findings can be generalized to a broader population. There are notable differences in walking speed between pre- and post-osseointegration. Walking speed is known to influence kinetics [36], and so, the observed kinetic differences might have also been influenced by the walking speed and not exclusively by osseointegration.

The stump, prosthetic liner, socket, and connector were combined to model the thigh as one rigid segment to reduce the computational complexity. This modelling approach did not incorporate the angular and axial movements at the socket-stump interface, which can be 12° and 36 mm [54]. Although small, the impact of these movements on the hip contact forces should be investigated. In the post-operative model, modelling the thigh as a rigid segment consisting of the stump and implant provides a reasonable approximation but does not capture the complex behaviour of the osseointegration interface, such as micro-motion effects, which are expected to be within any possible measurement errors. Moreover, the model assumed the external loads are transmitted axially through the distal end of the residual limb. While this accurately reflects cases involving end-bearing sockets, ischial containment sockets distribute the load more proximally to the ischium. This limitation may have impacted the pre-operative hip joint contact forces, as some force might have been transferred to the ischium instead. Future research could focus on modelling the force distribution through changes in the musculoskeletal model approach, which could be supplemented by finite element analysis. In the post-operative context, this limitation is mitigated by the absence of the socket, and the rigid coupling achieved through osseointegration ensures that the loads are directly transmitted through the upper leg to the hip.

Additionally, this study analysed gait only and did not include other activities of daily living, and gait alone does not represent overall musculoskeletal function [55]. Future research should analyse other movements.

## Conclusion

This study offers novel insights into the patient-focused outcomes and musculoskeletal system loading for persons with unilateral or bilateral transfemoral amputations who are not able to tolerate traditional suspension socket prostheses and have undergone osseointegration surgery. It underscores the post-operative improvements in functional abilities and more natural gait patterns in some cases. The diverse post-operative gait adaptations observed underscore the complexity of osseointegration surgery outcomes, emphasizing the value of in-depth case studies. Further research with larger cohorts and extended follow-up periods is necessary to comprehensively evaluate the implications of osseointegration on overall musculoskeletal function and mobility.

## Data Availability

No datasets were generated or analysed during the current study.

## Abbreviations

Q-TFA: Questionnaire for transfemoral amputations
UTF: Individuals with unilateral transfemoral amputations
BTF: Individuals with bilateral transfemoral amputations
OGAP-OPL: Australian Osseointegration Group of Australia-Osseointegration Prosthetic Limb
GRF: Ground reaction force
BW: Bodyweight
